# Eurytrematosis in cattle in southern Espírito Santo State, Brazil - case report

**DOI:** 10.29374/2527-2179.bjvm002023

**Published:** 2023-07-21

**Authors:** Renata de Paula Santos, Caio Alves Cardoso, Marcos Paulo Brinati Miranda, Eduardo Vargas de Oliveira, Júlio Francisco Valiati Marin, Natânia do Carmo Sperandio, Louisiane de Carvalho Nunes, Isabella Vilhena Freire Martins

**Affiliations:** 1 Undergraduate in Veterinary Medicine, Departamento de Medicina Veterinária, Centro de Ciências Agrárias e Engenharias (CCAE), Universidade Federal do Espírito Santo (UFES), Alegre, ES, Brazil; 2 Veterinarian, Programa de Pós-Graduação em Ciências Veterinárias, Departamento de Medicina Veterinária, CCAE, UFES, Alegre, ES, Brazil; 3 Veterinarian, DSc., Departamento de Medicina Veterinária, CCAE, UFES, Alegre, ES, Brazil.

**Keywords:** *Eurytrema coelomaticum*, epidemiology, pancreas, *Eurytrema coelomaticum*, epidemiologia, pâncreas

## Abstract

This work reports an outbreak of eurytrematosis in cattle in the municipality of Ibitirama, southern Espírito Santo State, Brazil. Six cattle were necropsied from August to December 2019, with finding of *Eurytrema coelomaticum* in the pancreas. A survey of epidemiological data was carried out on the farms along with coproparasitological examination of cattle from the same herd. Parasites were found in all necropsied animals, with different degrees of parasitism, ranging from mild to massive infection (6 - 2000 specimens). Macroscopic analyses of the pancreas revealed changes in 83.33% (5/6) of the cases, and by microscopy, pancreatic fibrosis ranging from Grade I to Grade III was observed. Inspection of the grazing areas confirmed the presence of two intermediate hosts, a terrestrial snail of the *Bradybaena* genus, with larval forms of the trematode in histological findings, and a grasshopper of the *Conocephalus* genus. Although none of the cattle showed clinical signs in the coproparasitological examination, 73.80% (31/42) tested positive for *E. coelomaticum* eggs. This is the first record of an outbreak of eurytrematosis in cattle in Espírito Santo State, indicating the importance of carrying out diagnosis based on epidemiology and necroscopic and parasitological examinations in animals in the region so that appropriate control measures can be adopted.

## Resumo

Este trabalho objetivou relatar um caso de euritrematose em bovinos no município de Ibitirama, Sul do Estado do Espírito Santo, Brasil. Foram necropsiados seis bovinos de agosto a dezembro de 2019, que apresentaram *Eurytrema coelomaticum* no pâncreas. Foi realizado levantamento de dados epidemiológicos nas propriedades e exames coproparasitológico em bovinos do mesmo plantel. Em todos os animais necropsiados foram encontrados parasitos, com diferentes graus de parasitismo, variando de infeção branda a maciça (6 - 2000 exemplares). Análises macroscópicas dos pâncreas revelaram alterações em 83,33% (5/6) dos casos e, na microscopia, observou-se fibrose pancreática variando de Grau I a Grau III. A inspeção das áreas de pastejo constatou a presença dos dois hospedeiros intermediários, moluscos terrestres do gênero *Bradybaena* com formas larvares do trematoda em achados histológicos e gafanhotos do gênero *Conocephalus*. Nenhum dos bovinos apresentou sinais clínicos, no entanto, no exame coproparasitológico, 73,80% (31/42) testaram positivo para ovos de *E. coelomaticum.* Este é o primeiro registro de surto de euritrematose em bovinos no estado do Espírito Santo, mostrando a importância da realização do diagnostico a partir da epidemiologia e de exames necroscópicos e parasitológicos em animais da região para que sejam adotadas medidas adequadas de controle.

## Introduction

Among the parasites that impact cattle breeding, the infection caused by flukes of the genus *Eurytrema* is particularly relevant due to its frequency in slaughterhouses in Brazil (Tessele et al., 2013). These parasites have wide cosmopolitan distribution in Brazil (Bassani et al., 2007). Some states in Brazil have been found to have high prevalence of the parasite, such as Paraná with 12.1%, Minas Gerais with 17.15%, and São Paulo with 80%. Studies in other countries, such as Malaysia, have found 97% of animals with alterations caused by the presence of the parasite. The high prevalence in some Brazilian states is due to factors related to the biological cycle of the trematode, particularities of each region and environmental conditions (Azevedo et al., 2004).

Animals with high parasitism rates have lesions that alter pancreas functions, and consequently interfere in digestion and food conversion. They cause clinical signs such as sudden cachexia, weakness and anemia, according to the degree of parasitism (Bassani et al., 2007; Headley et al., 2004). However, bovine eurytrematosis in most cases is subclinical in animals with good health condition, and only during slaughter or necropsy are the parasites detected (Bassani et al., 2007).

Although it can be considered a common condition in cattle in some regions of Brazil, recently published data on the prevalence are lacking, and there were no previous reports in Espírito Santo. This may be related to the subclinical nature, as described above, which makes it difficult to identify cases, causing underreporting of the disease and hampering the necessary control measures.

Here we report the presence or eurytrematosis in cattle in the region of Caparaó, in the south of Espírito Santo, to support decisions for implementation of control measures and prevention of new cases.

## Materials and methods

This study was carried out in the municipality of Ibitirama, located in the southern region of the state of Espírito Santo. We analyzed adult crossbred dairy cows, submitted to necroscopic examination performed at the Animal Pathology Laboratory of the Veterinary Hospital of the Center for Agrarian Sciences of Espírito Santo Federal University. The study was approved by the Ethics Committee on Animal Use - Alegre Campus (CEUA-Alegre, protocols 03/2017 and 025/2020).

The necropsies were performed according to the laboratory routine. Initially, the macroscopic and photodocumented alterations were examined, followed by the collection of tissue samples of significant lesions. The samples were fixed in 10% formaldehyde and sent to the Animal Pathology Laboratory for further histopathological processing and tissue staining with hematoxylin and eosin. The parasites found were collected and sent to Parasitology Laboratory of the same university.

We then returned to the farms to inspect the grazing areas and collect insects and snails, which were sent to the Parasitology Laboratory for identification and histological processing, according to the standard routine technique for these species of the Animal Pathology Laboratory. For the processing of the snails, initially they were placed in warm water, separated from the shell and fixed in 10% formaldehyde for 48 hours, then embedded in paraffin, processed according to the routine technique for this species and stained with hematoxylin and eosin.

Fecal samples were collected from live cows and sent to the Parasitology Laboratory where they were evaluated according to the specific sedimentation technique to detect eggs of *Eurytrema* sp., as described by Ueno and Gonçalves (1998), with reading of 5 slides of each sediment sample at 200x magnification.

Statistical analysis involved calculation of descriptive statistics (percentages and frequency and intensity of parasitism) and classification of degrees of pancreatic injury based on semi-quantitative subjective scores.

## Results

**Figure 1:**
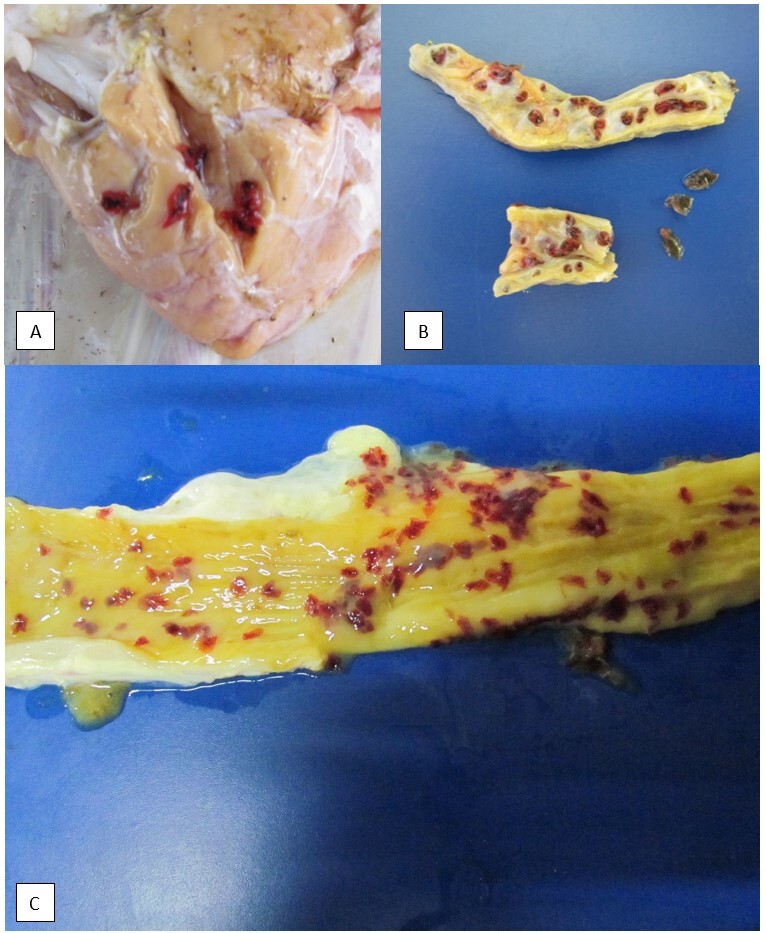
Photomacrographs of bovine pancreas samples with eurytrematosis from cows in the municipality of Ibitirama, ES, necropsied from August to December 2019. A) Pancreas revealing a pale color, with increased volume, containing numerous parasites in the shape of reddish leaves; B) Cross-section of the pancreas revealing dilation and fibrosis of the pancreatic ducts and the presence of parasites in the lumen; C) Cross section of the duodenum revealing the presence of parasites in the lumen.

Of the six necropsied cows, four came from the same farm and two to a neighboring property. The body condition score ranged from 1.5/5 to 3/5. Necroscopic examination of all animals revealed specimens of flattened parasites with a leafy appearance, compatible with the genus *Eurytrema*, found in the pancreas, with intensity varying from 6 to 2000 specimens. In 83.33% (5/6) of the cases, there were macroscopic lesions in the pancreas, and 50% (3/6) had diffuse thickening of the ducts, and of these, 33.33% (1/3) also had fibrosis of the pancreatic ducts and 33.33% (1/3) had associated intense diffuse peripancreatic edema. A color change was also observed in the pancreas of 33.33% (2/6) of the animals, one with a yellowish appearance and the other pale white, and 16.66% (1/6) showed an increase in volume. In addition to the pancreas, in 50% (3/6) of the cows, the parasite was observed in the initial portion of the duodenum (Figure 1).

Lesions in other organs were also observed, such as hepatomegaly associated with areas of pale white or yellowish areas in 83.33% (5/6) of the cases, areas of multifocal fibrosis on the visceral surface of the liver in 83.33% (5/6), and an increase in the size of mesenteric lymph nodes in 83.33% (5/6) of the animals.

In the histological analysis of the lesions caused by this parasite, 50% (3/6) of the cases presented hyperplasia and fibrosis of the pancreatic ducts, 16.66% (1/6) hyperplasia of the pancreatic ducts, 16.66% (1/6) fibrosis and hyperplasia of the pancreatic ducts associated with moderate diffuse inflammatory infiltrate and 16.66% (1/6) showed no alterations. Moderate to intense presence of parasites and trematode eggs were observed in all the pancreas fragments.

**Figure 2:**
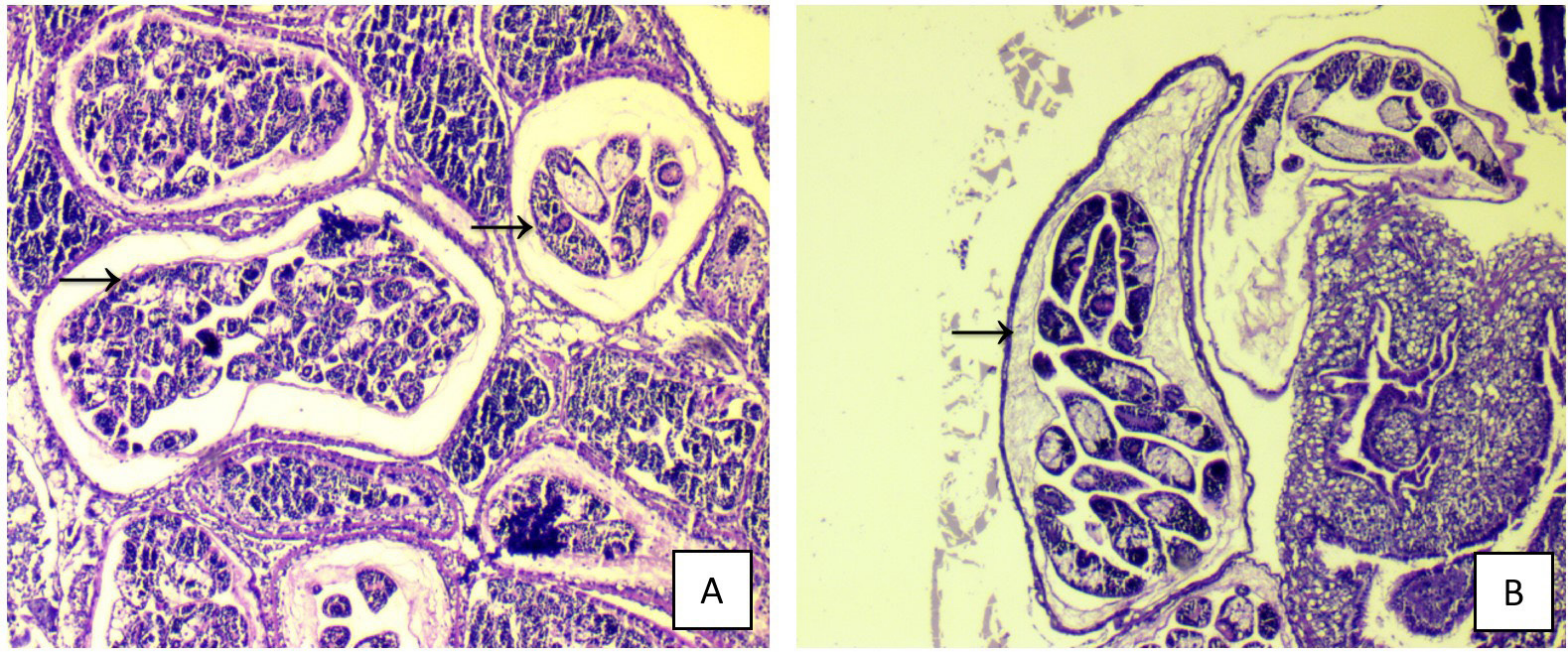
Photomicrograph of specimens of naturally infected *Bradybaena similaris* found in the grazing areas of farms with outbreak of bovine eurytrematosis in the municipality of Ibitirama, ES, between August and December 2019. Several sporocysts of *Eurytrema* can be observed in different stages in the anterior region of the digestive tract: A) Free sporocysts of *Eurytrema* in the digestive tract of snails of the genus *Bradybaena* (arrows), 4x objective; B) Detail of the sporocysts inside the digestive tract (arrow), 10x objective. Hematoxylin and eosin staining.

The parasite found in the pancreas and duodenum was identified as *E. coelomaticum*. In the inspection of the grazing areas and handling of the animals in the farms, the presence of intermediate hosts was found. Pastures of *Brachiaria humidicola* were observed with the presence of snails of the *Bradybaena* genus [according Araújo (1989)] and grasshoppers of the genus *Conocephalus*. In the histological evaluation of the snails, the presence of sporocysts of trematodes in the digestive tube was also observed (Figure 2).

The coproparasitological examination of the animals revealed that of the 42 cows submitted to the examination, 73.81% (31/42) tested positive for the presence of *Eurytrema* eggs in the feces, while in 25.59% (11/43) it was not possible to detect the presence of eggs of this parasite through the sedimentation technique used.

## Discussion

The slaughtered animals did not show clinical alterations such as lethargy, depression, weakness or anemia, as described by Ilha et al. (2005) and Quevedo et al. (2013). However, the animals had low body scores, which might have been related to pancreatic lesions, leading to progressive weight loss. It is noteworthy that most reports in the literature are findings from slaughtered animals.

In the definitive host infection, the metacercariae released from the cysts in the duodenum migrate to the pancreas through the accessory ducts and are distributed in the pancreatic ducts (Ilha et al., 2005). We found parasites both in the pancreatic ducts and duodenum, possibly related to the type of migration of *Eurytrema* and the degree of parasitism, since most studies have only reported the occurrence of the parasites in pancreatic ducts and occasionally biliary ducts (Surian et al., 2022). However, Bassani et al. (2006) and Schwertz et al. (2015) reported the occurrence in the duodenum was rare.

Regarding the classification of lesions, the three types of lesions mentioned by Yamamura et al.(1995) were verified: Type I - Absence of macroscopic lesions in the ducts and parenchyma; Type II - Presence of macroscopic lesions only in the ducts; and Type III - Presence of macroscopic lesions in the ducts and parenchyma. The histological examination confirmed the alterations observed macroscopically, and in some cases subsequent microscopic examination revealed alterations not detected macroscopically. Changes similar to those described by several authors were found, such as hyperplasia of the pancreatic ducts, dilation of the ducts and ductal fibrosis, associated with the presence of fragments of both adult parasites and eggs (Bassani et al., 2006; Ilha et al., 2005; Quevedo et al., 2013).

Only one of the pancreas samples analyzed showed the presence of inflammatory infiltrate. According to the injury patterns described by Quevedo et al. (2013), the lesions were classified in the first stage, characterized by an inflammatory infiltrate with a predominance of macrophages, lymphocytes and eosinophils. In comparison with the ranking of Belém et al. (1992), according to the predominant lesions, the presence fit in grade III. There were also no alterations in the Islets of Langerhans in all pancreas samples with alterations, probably indicating that the pancreatic endocrine function was not altered. However, this hypothesis needs further analysis, since the animals were not evaluated in this regard. One of the animals did not present any macroscopic or microscopic alterations, despite the presence of parasites in the ducts.

In field research, snails were found close to the pastures, along with grasshoppers, which are intermediate hosts of this fluke. Their infection was confirmed by histological studies, as previously performed by Brandolini and Amato (2001), who described *Eurytrema* sporocysts in the *Bradybaena* digestive tract.

Considering that this disease has subclinical character, it is important to conduct regional surveys. In this study, neither the animals that died nor the living ones from the same herd showed clinical signs, but the living ones had high frequency of the parasite in the coproparasitological examination. Although this test revealed a large percentage of infected animals, it is generally a little used technique, and most positive cases are accidental findings (Belém et al., 1992). Furthermore, the elimination of eggs can be delayed by obstructions and changes in the pancreatic tissues, so the number of eggs eliminated with the feces is not necessarily proportional to the number of flukes in the organ (Yamamura et al., 1995). The technique used in this study, sedimentation, is recommended mainly to detect trematode eggs, since it is more sensitive for detection of heavier eggs according to Ueno and Gonçalves (1998).

## Conclusion

This study is the first report of cases of eurytrematosis in cattle in Espírito Santo and shows the importance of diagnosis based on epidemiology, necroscopic and parasitological examinations of animals, so that adequate control and prophylaxis measures can be adopted.
